# Risk factor analysis for delayed union after subtrochanteric femur fracture: quality of reduction and valgization are the key to success

**DOI:** 10.1186/s12891-019-2775-x

**Published:** 2019-08-31

**Authors:** Viola Freigang, Franziska Gschrei, Himanshu Bhayana, Paul Schmitz, Johannes Weber, Maximilian Kerschbaum, Michael Nerlich, Florian Baumann

**Affiliations:** 1 Department of Trauma Surgery, Regensburg University Medical Center, 93042 Regensburg, Germany; 2 Department of Orthopaedics, UCMS and GTB Hospital, Delhi, 110095 India

**Keywords:** Subtrochanteric femur fracture (SFF), Delayed union, Non-union, Pseudarthrosis, Risk factor analysis, Fracture healing

## Abstract

**Background:**

Subtrochanteric femur fractures (SFF) are uncommon, but have a high complication rate concerning non-union and mechanical complications. There is ongoing discussion about risk factors for delayed fracture healing after SFF. The purpose of this study was to evaluate potential risk factors for delayed fracture healing after SFF.

**Methods:**

This retrospective radio-morphometric case control study compares 61 patients after SFF in two groups (uncomplicated healing within 6 months postoperatively vs. delayed union) concerning radiographical properties. The patients were analyzed concerning the following parameter: Quality of the reduction according to Baumgaertner, CCD-angle, Tip-Apex Distance, leg-length shortening and fracture healing according to the RUSH Score.

**Results:**

The mean RUSH-Score at 6 months postoperatively was 21.32(±4.57). At that point of time, only 29/61 fractures were radiographically fully consolidated (timely fracture healing) and 32 patients were rated as delayed union. The total revision rate was 9/61 (14.7%), whereof four patients required revision for symptomatic non-union of the SFF. The results of the radio-morphometric measurement showed a significant difference between both groups concerning the degree of reduction measured according to Baumgaertner (*p* = 0.022). The postoperative ipsilateral CCD-angle was different between the two groups (*p* = 0.019). After 12 months postoperatively, 48/61 (78.6%) of fractures were rated healed without any further intervention.

**Conclusions:**

Delayed union after SFF occurs frequently. In our patient population, the quality of reduction and the postoperative CCD-angle were the key factors to avoid delayed union.

**Level of evidence:**

Level III, Therapeutic study.

**Trial registration:**

Clinical Trial Registry University of Regensburg Z-2018-1074-1. Registered 04. Aug 2018. https://studienanmeldung.zks-regensburg.de

## Introduction

Subtrochanteric femur fractures (SFF) are fractures of the trochanteric region involving the lateral trochanteric wall or involving the area 5 cm below the lesser trochanter [1, 2]. These injuries are less common, but have the highest mechanical complication rate among extraarticular proximal femur fractures [3]. The standard treatment for SFF is reduction and fixation with a cephalon-medullary nail or extramedullary with a plate [2, 4–15]. A SFF may lead to a substantial impairment of musculo-skeletal function and the patient’s health-related quality of life [16–18]. Leg-length shortening, delayed union, non-union and mechanical complications are typical complications of SFF [10, 12, 19–21]. Demographic changes in industrial countries with over-aging of societies and an increase of comorbidities will lead to increased numbers of proximal femur fractures and to more complicated cases [22]. In literature, there is ongoing discussion about risk factors for delayed fracture healing after SFF. Besides general risk factors like bone metabolic disorders (e.g. bisphosphonates, diabetes etc.), mechanical instability is seen as the most important specific risk factor for delayed fracture healing in SFF [10, 16, 17, 21].

Fracture healing is generally based on multiple clinical and radiological factors indicating a timely healing of the bone at fracture site. There are several terms being used to describe the status of fracture healing such as timely healing, delayed healing and non-union (describing a condition where the progression of the fracture healing has come to a halt before fracture consolidation). There is ongoing discussion about the definition of these terms [3, 23]. For most authors, a status where fracture consolidation has not occurred within the first 6 months after trauma is seen as delayed union [3, 22–25]. For our study, this definition was used as main criterion for evaluation of the fracture healing. [3, 23] For trochanteric fractures, there is clear data that quality of reduction and the position of the implant are crucial issues to prevent delayed union or mechanical complications [9, 22, 24–26]. Most studies dealing with proximal femur fractures report on SFF as a subtype of trochanteric fractures [24, 26, 27]. This led to the assumption that the same risk profile can be applied to SFF. Therefore, factors like the Tip-Apex-Distance, the Cleveland Index, and quality of reduction are seen as risk factors for delayed fracture union in SFF as well [24, 28]. However, there is not enough evidence in literature supporting this assumption. There are some studies advocating for an anatomic reduction of the fragments and the use of cerclage wires [5, 12, 16, 29]. The question remains if the construct of stable cephalic fixation measured by the Tip-Apex-Distance and the Cleveland-Index is applicable to SFF where in most cases the cephalo-trochanteric integrity is not disrupted. Clinical failure analyses of delayed union after SFF show that the mechanical failure does not occur as cut-out like in trochanteric fractures, but in a breakage of the implant at former fracture site in the subtrochanteric region [10, 19, 21, 25]. Biomechanical studies have shown that due to lack of the lateral cortical support in SFF, the large lever arm of the femur leads to mechanical instability of the subtrochanteric region [29, 30]. Accordingly, the crucial point for timely fracture healing might be an increase of stability leading to a reduction of transmission forces of the subtrochanteric region.

The purpose of this study was to evaluate potential risk factors for development of a delayed fracture healing after SFF using a radio-morphometric analysis. Our hypothesis was that patient-related factors like comorbidity and surgeon-related factors like quality of reduction and positioning of the implant would have an impact on fracture healing.

## Materials and methods

The Ethics Committee at the University of Regensburg approved the study in August 2018 (Institutional Review Board Number 18–1130-104). The study is registered at the Clinical Trial Registry University of Regensburg Z-2018-1074-1. All procedures performed in this study were in accordance with the 1964 Helsinki declaration.

### Baseline characteristics

This study is a retrospective radio-morphometric case-control study to evaluate potential risk factors for development of delayed union after SFF. We reviewed the electronic medical database of our institution to identify all patients with SFF from 01/2007–12/2017. Of 121 cases with intramedullary nailing for SFF, 60 were excluded: 9 patients died within the first 3 months after injury, 39 patients were not willing to participate in the study or were excluded for lost to follow-up, 12 patients were excluded for a pathologic fracture leaving 61 patients with a complete dataset for further analysis. The mean age of the patients was 58.8 years (±18.4). There were 39 male (63.9%) and 22 female (36.1%) patients. 36/61 patients (59.0%) sustained a high energy trauma, whereas in 25/61 (41.0%) a simple fall was the reason for the SFF. All patients were treated according to the same treatment protocol with immediate partial weight bearing for 6 weeks postoperative and full weight-bearing after 6 weeks. Radiographic controls were made at 6 weeks, 12 weeks, and then every 3 months until consolidation of the fracture. Fracture healing was rated using the Radiographic Union Scale for Hip (RUSH Score) [31]. With a RUSH-Score greater than 22, the fracture was considered healed. The radiographic measurements were performed by VF and FG under supervision of FB. Minimum follow up was 12 months postoperatively. Data were acquired from the institution’s database. All patients were contacted for their informed consent and to investigate if the patient had been treated elsewhere. For these cases, all co-treating institutions were contacted and information transferred electronically. The patients were divided in two groups: Group I with uncomplicated fracture healing within 6 months after surgery and group II with no radiographic fracture healing within 6 months after surgery (delayed fracture healing).

### Radio-morphometric analysis

The fracture type was classified in preoperative radiographs (pelvis a.p. and axial view of the hip) using the Seinsheimer Classification [2].

Postoperative x-rays were used for the risk-factor analysis. The x-rays were blinded and processed for the software-based digital analysis using the mediCAD planning software (mediCAD-Software, Version 5, Hectec Inc., Altdorf, Germany). To eliminate measurement errors due to magnification of the x-ray images, we adjusted every image referencing to the largest diameter of the implant (e.g. proximal diameter of the cephalo-medullary nail).

**Fig. 1:**
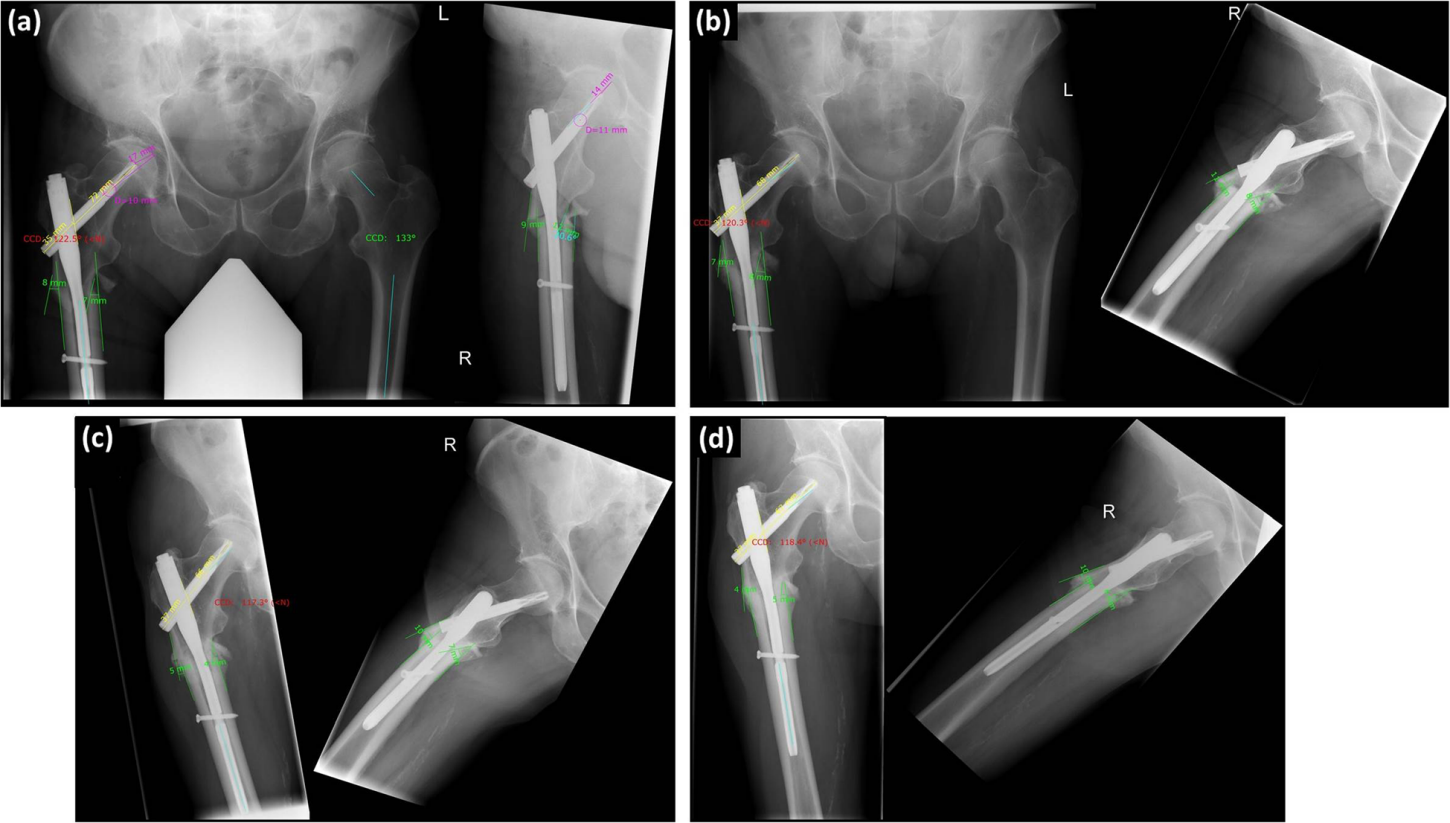
Radio-morphometric measurement and course of healing after subtrochanteric femur fracture [Legend of measurements: Tip-apex distance (purple), Sliding distance of the femoral neck blade (yellow), distance of the fragment dislocation (green), CCD-angle measurement in degree (measurement = light blue, result = red)]

**Fig. 2:**
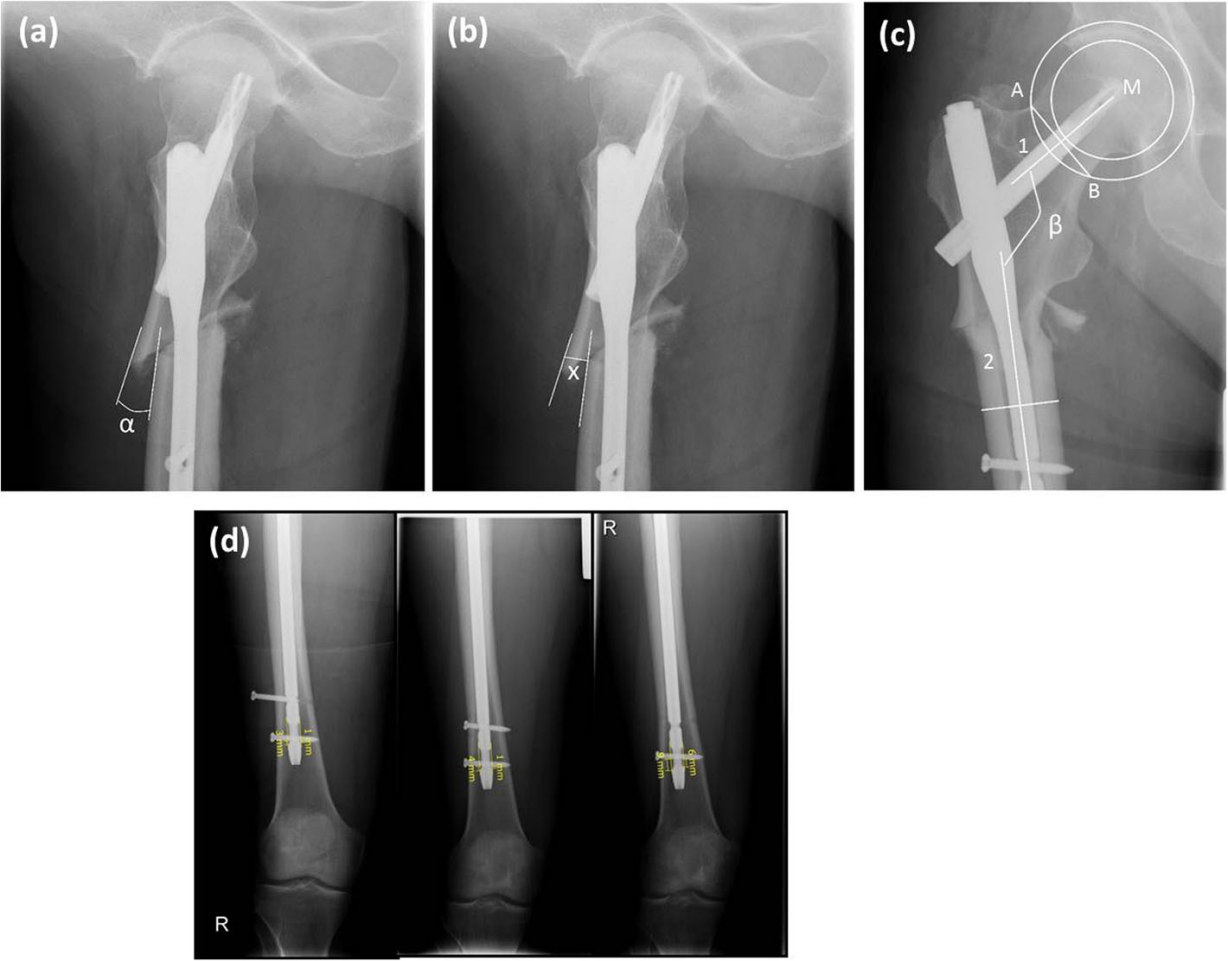
**a** Postoperative lateral radiograph measuring postoperative shaft angle (α) at fracture level. **b** Postoperative lateral radiograph measuring ventral (x) dislocation at fracture level. **c** Measurement of the CCD-angle: M = center of the femoral head, A = lateral point at the narrowest part of the femoral neck, B = point at medial femoral neck (through 2nd arc of a circle), 1 = femoral neck axis (vertical reference line to A-B through M), 2 = femoral shaft. **d** Anterior-posterior radiographs at 6 weeks, 3 months (after change of distal locking bolts) and 6 months postoperative: Radiographic measurements of the leg length shortening

The following parameter were recorded (Fig. 1):

- Singh Index for bone quality [32]
- Quality of the reduction according to the Baumgaertner classification system [24]
- Postoperative shaft angle at fracture level in ° (α): Application of a straight line at the cortical bone of the proximal and the distal fragment and measurement of the angle with respect to these two straight lines (Fig. 2a)
- Ad latus dislocation at fracture level in mm (x = ventral and y = lateral): Application of a straight line at the ventral (x) and lateral (y) cortical bone of the proximal and the distal fragment. The amount of dislocation is the gap between these two straight lines measured in mm at the point of the proximal end of the distal fragment (Fig. 2b).
- Centrum-collum-diaphyseal (CCD) angle of ipsilateral and contralateral side in ° (β): Measurement of the CCD-angle on an a.p. radiograph
  1. Appointing the center of the femoral head with a circle template: the lateral part of epiphysis and the medial angle of the femoral neck serve as reference points for the arc of circle.
  2. One point is marked at the lateral end of the narrowest part of the femoral neck.
  3. Another arc of a circle passing through this point is constructed with the femoral head as center.
  4. The intersections of the second circle and the femoral neck are connected.
  5. The femoral neck axis is the vertical reference line to that straight line through the center of the femoral head.
  6. The longitudinal axis of the femoral shaft is the center line between the contours of the femoral shaft (Fig. 2c).
- Tip-Apex Distance in mm [24]
- Fracture healing using the RUSH Score [31]
- Leg-length shortening in mm (Fig. 2d)

Besides the radio-morphometric analysis, clinical parameters; comorbidities, medication and revisions were recorded.

### Statistical analysis

Statistical analysis was performed using the software package SPSS (Version 25, SPSS Inc., Chicago, Illinois). For comparison of mean values, we used the independent t-test. For ordinal data, we used the Chi-square test. We used the Mann-Whitney-U-Test for non-parametric comparison testing. Unless otherwise stated, descriptive data are given as mean ± standard deviation. The level of significance was at *p* < 0.05 for all tests.

## Results

Table 1 shows baseline characteristics and radio-morphometric measurement results recorded in 61 patients. According to Seinsheimer, there were no type I injuries, 10 type II (2xA, 6xB, 2xC), 19 type III (17xA, 2xB), 13 type IV, and 19 type V injuries. No open fractures occurred. The patients had surgery on average 0.59 days after the injury (±1.36). An open reduction was necessary in 35/61 patients (57.4%). The surgeon used cerclage wires in 32/61 (47.5%) of the cases. In 56/61 patients a PFN-A (Synthes Inc., Zuchwil, Switzerland) was used for osteosynthesis (short PFN-A in 16 patients, long PFN-A in 40 patients). An AFN was used in 2/61 and a Gamma Nail (Stryker Inc., Kalamazoo, USA) was used in 3/61. Four patients (5.5%) had a concomitant compartment syndrome of the thigh treated by fasciotomy and secondary closure within 10 days at the latest. 6/61 (9,8%) showed prolonged wound healing with secretion longer than day 5, none of them needed revision for surgical site infection. Two patients (2.7%) were revised for malreduction concerning rotation within the first week after initial surgery. During the follow-up period, 3/61 sustained a mechanical complication (2 patients breakage of the implant 4 months and 6 months after initial treatment, 1 patients with adjacent fracture 6 weeks after). Six months postoperatively, only 29/61 (47.5%) fractures were radiographically fully consolidated (timely fracture healing). According to the definition, 32/61 patients were rated as delayed fracture healing. The mean RUSH-Score at that point of time was 17.6 (±2.34). We did not see any discrepancy between the RUSH Score and the clinical experience-based evaluation. Clinical experience-based evaluation means secondary signs of instability e.g. implant failure or signs of lysis around the implant.

**Table 1:** Baseline Characteristics and radio-morphometric measurement results

*N* = 61	Group I (uncomplicated healing)			Group II (delayed union)			Level of significance	Total
Age at time of injury in years	54.27 ± 16.34			58.51 ± 20.06			*p* = 0.381	56.7 ± 18.54
BMI at time of injury	26.9 ± 4.1			28.42 ± 6.65			*p* = 0.336	27.79 ± 5.75
Grade of Quality of reduction (Baumgaertner)	I	II	III	I	II	III	*p* = 0.022^*^	
	30.8%	50.0%	19.2%	11.4%	60.0%	28.6%		
Postoperative ventral dislocation of the proximal fragment in mm (x)	5.5 ± 5.7			7.74 ± 5.48			*p* = 0.155	6.27 ± 5.39
Postoperative lateral dislocation of the proximal fragment in mm (y)	6.62 ± 6.83			6.0 ± 4.05			*p* = 0.686	6.12 ± 5.40
Ipsilateral CCD-angle (β) (postoperative)	127.46 ± 6.24			123.23 ± 7.05			*p* = 0.019^*^	125.06 ± 6.98
Contralateral CCD-angle in ° (β‘)	131.21 ± 5.91			128.66 ± 6.06			*p* = 0.106	129.75 ± 6.09
Tip-Apex-Distance postoperative in mm	24.87 ± 9.37			28.81 ± 13.91			*p* = 0.256	27.14 ± 12.25
RUSH-Score at 6 months postoperative	25.15 ± 2.78			17.63 ± 2.34			*p* = 0.001^*^	21.32 ± 4.57
Leg-length shortening at 6 months postoperative in mm	1.06 ± 1.3			4.07 ± 8.14			*p* = 0.193	2.42 ± 5.65

* significant at level *p* ≤ 0.05

Four patients required revision for symptomatic non-union of the SFF. Accordingly, the total revision rate was 9/61 (14.7%). The mechanism of injury (low-energy vs. high energy trauma) did not differ between the “uncomplicated healing” and “delayed union” group (*p* = 0.400) (Table 2). Mean time from surgery to complete fracture consolidation for all patients was 8.0 months (±7.7 months, range 1.4–28.9). After 12 months 48/61 (78.6%) of patients were rated as healed without any further intervention.

**Table 2:** Healing rates in relation to trauma mechanism

*N* = 61	Group I (uncomplicated healing)	Group II (delayed union)	Total
High-energy trauma	15 (41.7%)	21 (58.3%)	36 (59.0%)
Low-energy trauma	14 (53.8%)	11 (46.2%)	25 (41.0%)
Total	29 (47.5%)	32 (52.5%)	61 (100%)

The results of the radio-morphometric measurement showed a significantly greater CCD-angle and a higher degree of reduction measured according to Baumgaertner for the “uncomplicated healing” group (*p* = 0.022) [24]. There was a significant difference for the postoperative ipsilateral CCD-angle (*p* = 0.019) but not for the uninjured contralateral side (*p* = 0.106). This finding is in accordance with previous findings in biomechanics that a lower CCD-angle increases mechanical load of the subtrochanteric region [6, 31, 33]. The impact of a residual postoperative dislocation in ventral direction had a greater impact than lateral ad latus dislocation; however, both not significant (*p* = 0.155, *p* = 0.686). The delayed union group had a greater value for radiographic leg-length discrepancy after consolidation, however, this was not significant (*p* = 0.193). The groups were not different concerning the position of the cephalic blade measured by the Tip-Apex-Distance or the Cleveland Zone. The degree of osteoporosis evaluated by the Singh-Index was not different for the two groups.

There was no significant difference between the two groups concerning general comorbidities like prior neoplastic disease, diabetes, peripheral artery disease, osteoporosis, nicotine or alcohol abuse. Prior medication (anticoagulation, steroids, chemotherapy, or bisphosphonates) had also no significant impact on delayed healing.

## Discussion

The most important finding of the present study is that the CCD-angle and the quality of reduction are key factors for fracture healing in SFF. These factors underline the importance for mechanical stability in case of a fracture of the subtrochanteric region. The rate of delayed union was as high as 52.5%. However,12 months postoperatively 48/61 (78.6%) of fractures were healed without any further intervention. In contrast to other studies reporting on trochanteric and subtrochanteric fractures, we did not see any correlation between fracture healing and cephalic position of the implant measured by the Tip-Apex Distance or the Cleveland zone of the cephalic blade [24, 25]. To our knowledge, this is the largest study investigating radiographic fracture healing of patients after intramedullary nailing for SFF.

Demographic changes will lead to a rising number of SFF over the next decade [22]. The distribution of age for this injury is bimodal: younger patients sustaining a high velocity trauma and geriatric patients with a SFF after low energy trauma like a simple fall. Increasing numbers can be expected especially in older patients. This older growing patient population has a high level of comorbidity [17, 18, 22]. SFF frequently show a prolonged fracture healing. Younger patients have a greater compensatory potential, whereas older patients are life-threatened by immobilization in case of a proximal femur fracture. In our study population, the patients with prolonged healing had a higher mean age; however, this was not significant. Other studies reported on patients with a higher average age [6, 12, 16–18]. In literature, age is seen as general risk factor for delayed fracture healing [6, 26, 31, 33]. Regarding comorbidities, our study revealed no significant patient-related risk factors for delayed fracture healing after SFF. However, we report on a small number of patients. An association between fracture healing and these potential risk factors cannot be rejected due to low power of the study.

These general risk factors are not influenceable in case of an acute event of a fracture. In contrast, specific risk factors can be modulated. In literature, there is a controversial debate about potential risk factors for prolonged fracture healing in SFF. Main problem is that there are no large series since SFF is an uncommon injury. There is quite a number of studies dealing with larger patient populations on proximal femur fractures in general [9, 22, 26, 27]. Most of these studies report on SFF as a subgroup of trochanteric fractures and include only a small number of SFF. However, SFF, trochanteric fractures, or femoral neck fractures have completely different biological and biomechanical set of properties [6, 9, 12, 16–18, 22, 26, 27]. Therefore, it is reasonable to investigate this type of fracture separately.

The subtrochanteric region transmits great forces that arise from the long lever arm of the femoral shaft. The lateral wall has to restrain tractive forces whereas the medial buttress has to bear compressive stress [4, 11, 29, 33]. Anatomical reduction (instead of malalignement) and fragement compression may reduce the forces on the fracture site. Prior studies by Shukla et al. [12] and Codesido et al. [16] have shown that the quality of reduction has a large impact on fracture healing in SFF. Moreover, an interfragmentary compression of fragments applied by cerclage wires can improve stability of the subtrochanteric region [29] and lead to shorter time to healing and improved functional outcomes [16, 25]. In this radio-morphometric study, we used the classification system by Baumgaertner, which was developed to assess the quality of reduction in trochanteric fractures and adopted for SFF [24]. Comparing the delayed union group with the uncomplicated healing group, we found significantly lower values of the Baumgaertner classification indicating a less accurate degree of reduction than in the control group. This is concordant to prior studies on the relationship of healing rate and quality of reduction [5–7, 10–12, 14–16, 25, 34]. For the reduction of the fracture reconstruction of the femoral axis including the centrum-collum-diaphyseal (CCD) angle is important. From a biomechanical point of view, a reduced CCD angle indicating a varus deformity increases the load of the medial wall in the subtrochanteric area. Therefore, reconstruction of the medial cortex is one of the main treatment goals in surgical management of SFF. Our study is the first to prove a relationship of the CCD angle and delayed union in SFF. According to biomechanical studies, the CCD-angle seems to be a risk fracture for increased mechanical load of the subtrochanteric region leading to prolonged fracture healing [6, 26, 31, 33].

We found an exceptionally high rate of radiographically delayed union of 52.5%. At first sight, there is no obvious reason for this high number. Comparable studies did not provide separate data on fracture healing after 6 months. Thus, the mean healing times they reported (6.1–6.9 months [6, 12, 16]) indicate that a delayed union rate around 50% is not unusual in SFF. With 8 months, the mean healing time in our patient population was marginally longer. The evaluation of the progress of fracture healing was not only based on clinical experience, but we used the RUSH-Score in addition to determinate fracture healing. A score greater or equal 22 was rated healed [31].

The question remains if this high delayed union rate is clinically relevant. After 12 months postoperatively, 48/61 (78.6%) of fractures were healed without any further intervention. And there were only 9.6% of patients who required revision surgery for non-union after SFF. This indicates that prolonged fracture healing is a common phenomenon but of less clinical importance than in other injuries. Nevertheless, the surgeon should aim for greatest mechanical stability to prevent implant loosening or breakage of the implant before regaining intrinsic bony stability by completion of the fracture healing. The rate of mechanical complications was lower than in other studies in literature [12, 16]. Thus, mechanical complications remain a clinically relevant issue. It was not part of this study to evaluate the impact of prolonged fracture healing on the health-related quality of life (HR-QoL). Prior studies on subtrochanteric fractures have addressed this issue and found a relevant impairment of HR-QoL [6, 16]. In a prospective trial on patients aged over 60 years, Codesido et al. [16] reported an EQ-5D Index of 0.61 in 60 patients with subtrochanteric fragility fractures after 6 months. They found a relevant increase of the HR-QoL after 12 months.

Our study has some limitations. Surely, the main limitation is the retrospective design and the limited number of cases in our study. SFF are uncommon injuries making it difficult to conduct a prospective study with an adequate number of patients. However, this is the largest series of patients investigating radiographic fracture healing after SFF. Methodically, the radio-morphometric analysis has also some limitations. We used a planning software-based approach to minimize measurement errors caused by magnification of plain radiographs. A CT-based approach would have been more accurate; however, the load of radiation would not have been justifiable under study conditions.

## Conclusion

The most important finding of the present study is that the postoperative CCD-angle and the quality of reduction are key factors for fracture healing. The rate of delayed union in our study population was high (52.5%), however, at 12 months postoperatively 48/61 (78.6%) of fractures were healed without any further intervention. In conclusion, we advocate for anatomical reduction and valgization of the proximal femur in surgical treatment of SFF to avoid prolonged fracture healing.

## Data Availability

Please contact the author for data requests.

## Abbreviations

CCD: Centrum-collum-diaphyseal
HR-QoL: Health-related quality of life
PAD: Peripheral arterial disease
RUSH Score: Radiographic Union Scale for Hip
SFF: Subtrochanteric femur fractures
TAD: Tip-Apex-Distance
